# Healthcare utilization and productivity losses associated with CIDP: results from an international survey

**DOI:** 10.3389/fneur.2026.1846192

**Published:** 2026-07-01

**Authors:** Febe Brackx, Clémence Arvin-Berod, Sandra Paci, Johanna Maria Van Dongen, Judith E. Bosmans, Yasmin Taylor, Jack Wright, Gabi Faust, Sarah Dewilde

**Affiliations:** 1 Services in Health Economics, Brussels, Belgium; 2 Argenx, Ghent, Belgium; 3 Department of Health Sciences, Faculty of Science, Vrije Universiteit Amsterdam, Amsterdam Public Health Research Institute, Amsterdam, Netherlands; 4 Adelphi Real World, Bollington, United Kingdom; 5 GBS CIDP Selbsthilfe NRW, Neuss-Uedesheim, Germany

**Keywords:** burden of disease, caregiver burden, chronic inflammatory demyelinating polyneuropathy, CIDP, HCRU, productivity losses, resource utilization

## Abstract

**Introduction:**

Chronic Inflammatory Demyelinating Polyradiculoneuropathy (CIDP), a rare autoimmune disorder affecting peripheral nerves, is associated with substantial societal costs. This study assesses healthcare utilization and productivity losses across varying levels of patient disability.

**Methods:**

Data were obtained from Adelphi’s CIDP Disease Specific Programme™ (September 2022–April 2023), a digital, international, real-world survey involving neurologists and their patients (UK, France, Germany, Italy, Spain). Disability was measured using the Inflammatory Neuropathy Cause and Treatment (INCAT; range: 0–10), and categorized as mild (≤2), moderate (3–4), or severe (≥5). Neurologists provided data on treatment, informal care, hospitalizations, mobility aids, and home modifications for 542 patients, while lost productivity was reported by a subset of patients (*n* = 199). Associations between disability level and healthcare utilization and lost productivity were evaluated using linear regression, adjusting for age and time since diagnosis.

**Results:**

Of the 542 patients, 236 (43.5%) had mild, 189 (34.9%) moderate, and 117 (21.6%) severe disability. Treatment rates increased with severity (receiving treatment: mild 78.4%, moderate 89.9%, severe 92.3%; *p* < 0.001). The proportion of patients receiving informal care rose sharply with increasing disability (mild 7.2%, moderate 31.2%, severe 62.9%; *p* < 0.001). Hospitalizations in the 12 months prior to the survey were more frequent with greater disability (mild 7.0%, moderate 14.5%, severe 21.6%; *p* < 0.001), as were emergency admissions (1.0, 9.9, 13.7% respectively; *p* < 0.001) and ICU stays (0.0, 0.7, 3.9% respectively; *p* = 0.001). The use of mobility aids (19.1, 56.1, 89.7% respectively) and home modifications (12.8, 45.8, 74.2% respectively) also rose significantly with severity (*p* < 0.001 for both). Finally, work productivity losses among employed patients and activity impairment rose as disability increased (average percentage of productivity loss: mild 21.8%, moderate 44.0%, severe: 67.3%, *p* < 0.001; average daily activity impairment: mild 28.6%, moderate: 44.6%, severe: 68.1%, *p* < 0.001).

**Conclusion:**

CIDP is associated with substantial healthcare utilization that increases with increasing disability, highlighting the progressive functional decline associated with more severe disease. In addition to direct healthcare demands, CIDP imposes considerable caregiver support needs, changes in living conditions, and productivity losses. Together, these findings underscore the wide-ranging impact of CIDP and the need for comprehensive management strategies.

## 1 Introduction

Chronic inflammatory demyelinating polyradiculoneuropathy (CIDP) is a rare, immune-mediated neuropathy, characterized by progressive weakness and sensory dysfunction (1, 2). Symptoms generally develop gradually over more than 8 weeks, though a faster onset within 4 weeks occurs in up to 13% of cases (3). Treatments like intravenous immunoglobulin and corticosteroids aim to control symptoms and induce remission. However, despite treatment, many patients continue to face a considerable disease burden and often experience physical limitations and fatigue that hinder their ability to work and carry out daily activities (2, 4–6). Moreover, the emotional and social consequences of CIDP, such as anxiety, depression, and strained interpersonal relationships, further contribute to the overall disease burden (2, 4, 5).

Multiple studies have also demonstrated that CIDP is associated with a substantial economic burden (7–10). Immunomodulatory treatments accounted for a significant portion of total healthcare costs across studies (8, 10), while other societal costs were primarily attributable to productivity losses (8, 10). However, these studies were generally limited by small sample sizes and single-country settings, and none of them accounted for caregiver burden, even though informal caregiving entails considerable costs, and its exclusion may lead to an underestimation of the total economic impact of a disease (11, 12).

This study aims to document healthcare resource utilization (HCRU) associated with CIDP, including treatments, hospitalizations, mobility aids, home modifications and caregiver burden, as reported by neurologists in a large international survey. As a secondary aim, we will report employment rates and productivity losses in a subset of the patients for whom patient-reported data is available. These findings provide real-world evidence on the burden of illness in CIDP, highlighting areas of unmet need and supporting decision-making regarding resource allocation and treatment strategies. They also serve as valuable input for economic models evaluating the cost-effectiveness and budget impact of treatments for CIDP.

## 2 Methods

### 2.1 Study design and setting

This study utilized data from the Adelphi Real World CIDP Disease Specific Programme™ (DSP™), a cross-sectional survey that included both physician and patient inputs. Conducted between September 2022 and April 2023, the study spanned five European countries: France, Germany, Italy, Spain, and the United Kingdom. Physicians were selected through regional fieldwork agencies and were eligible if they specialized in neurology and typically managed at least two patients with CIDP per month. Patients qualified for participation if they were 18 years or older with a neurologist-confirmed diagnosis of CIDP.

Participating neurologists completed patient record forms (PRFs) for two to ten of their most recently seen CIDP patients. PRFs primarily captured point-in-time data, but also incorporated information from patients’ medical records, such as treatment history from diagnosis to the present, adding a retrospective element of data collection. These same patients were invited to independently and voluntarily complete a patient self-completion survey (PSC). PSCs contain self-reported data on the patient’s experiences with the condition, as well as validated patient-reported outcome measures (PROMs), such as the Work Productivity and Activity Impairment (WPAI) questionnaire. The overall methodology of the DSP has been described (13, 14), validated (15), and shown to be reliable and representative over time (16). Ethical exemption was granted by the PEAL Institutional Review Board (protocol #22-ADRW-153).

### 2.2 Collected data

Neurologists (*n* = 83) completed the PRF for a total of 542 patients. Key demographic information, such as age, sex, and country of residence was recorded. CIDP subtype was also documented and included typical CIDP as well as five variants: distal CIDP, multifocal CIDP, focal CIDP, motor CIDP, and sensory CIDP. Patients’ disability level was measured using the Inflammatory Neuropathy Cause and Treatment (INCAT) score. This scale includes both arm and leg disability, with a total score ranging from 0 (no impairment) to 10 (complete loss of purposeful movement) (17). HCRU was assessed across different domains: treatment, assessments used for monitoring patients’ CIDP, informal (unpaid) caregiving and professional caregiving, hospitalizations, mobility aids and home modifications. For treatments, neurologists reported the maintenance treatments prescribed at the time of the survey, the line of treatment, whether the patient had ever received rescue/acute treatment for their CIDP and the type of rescue/acute treatment. The line of treatment refers to the sequential order of therapies a patient is prescribed: first-line is the initial therapy, second-line the subsequent therapy given after discontinuation or change, and so forth. For assessments/tests used to monitor patients’ CIDP, neurologists reported which assessments were used and how many assessments were performed in the 12 months prior to the survey. Additionally, neurologists provided information on whether the patient required caregiver support, the average number of caregiving hours per week, the caregiver’s relationship to the patient, and the impact of caregiving on the caregiver’s daily activities and employment. They also reported the number of hospitalizations in the 12 months prior to the survey, including the number of nights spent in the hospital, admissions through the emergency room (ER) and intensive care unit (ICU) stays. Finally, neurologists indicated the mobility aids and home modifications needed by the patient. Data on visits to healthcare practitioners were missing for the majority of patients and were therefore not included.

Of the 542 patients, 199 completed the PSC. The PSC included a question on current employment status and the WPAI questionnaire, a validated tool used to measure the impact of health problems on an individual’s ability to work and perform regular daily activities (18). It quantifies absenteeism (percentage of work time missed), presenteeism (percentage of impairment while at work), overall work productivity loss (combined percentage of absenteeism and presenteeism), and activity impairment (percentage of impairment in daily activities) over the past 7 days.

### 2.3 Statistical analyses

Given the established association between functional disability and both healthcare and productivity costs (10), HCRU was reported according to disability level, as measured by the INCAT score. As no validated cut-off points of the INCAT score have been established, the following categories were used: mild disability (score ≤ 2), moderate disability (score 3–4), and severe disability (score ≥ 5). These same categories were used in a cost of illness study in Germany (10). Categorical variables were presented as frequencies and percentages, while continuous variables were summarized using the mean, standard deviation (SD), median, and interquartile range (IQR). Descriptive statistics were calculated using the available observations, with the corresponding sample size reported for each variable. We assessed the association between disability level and continuous variables using linear regression, or logistic regression for binary variables. Age and time since diagnosis were included as covariates to adjust for differences between groups related to age and time since diagnosis. Disability level was modeled as a categorical variable, with *p*-values reflecting the joint effect of the two dummy variables representing disability severity. To account for clustering of patients within neurologists, a random effect for neurologist was included. The mean number of assessments, caregiving hours, hospitalizations, and hospital nights were calculated for the entire population, with values of 0 assigned to patients who did not require caregiving or were not hospitalized. WPAI outcomes were presented for patients of working age (18–64 years). In addition to the analyses by severity level, the main outcomes were also evaluated separately for patients with typical CIDP and CIDP variants.

## 3 Results

### 3.1 Patient and disease characteristics

Patient and disease characteristics are summarized in Table 1. The sample comprised a total of 542 patients, including 236 (43.5%) with mild disability, 189 (34.9%) with moderate disability, and 117 (21.6%) with severe disability. Across all disability levels, the majority of patients were male (mild: 61.4%; moderate: 60.8%; severe: 65.8%) and presented with typical CIDP (mild: 64.8%; moderate: 69.8%; severe: 70.1%). The mean (SD) age was 54.0 (12.4) years and increased with disability severity, from 50.9 (12.1) years in the mild group to 55.5 (12.5) in the moderate group and 57.9 (11.1) in the severe group (*p* < 0.001). Similarly, the median (IQR) time since diagnosis increased across disability levels: 27.5 (42.6) months in the mild group, 39.8 (55.9) in the moderate group, and 46.5 (52.5) in the severe group (*p* = 0.003). The median number of patients per neurologist was 6 (Q1–Q3: 6–10). The patient and disease characteristics for the subset of patients for whom patient-reported data is available is reported in Supplementary Table A1.

**Table 1:** Patient and disease characteristics.

Characteristic	Category	Total (*N* = 542)	Mild disability (*N* = 236)	Moderate disability (*N* = 189)	Severe disability (*N* = 117)	*p*-value
Sex	Female Male	205 (37.8%) 337 (62.2%)	91 (38.6%) 145 (61.4%)	74 (39.2%) 115 (60.8%)	40 (34.2%) 77 (65.8%)	*p* = 0.653
Age	Mean (SD) years	54.0 (12.4)	50.9 (12.1)	55.5 (12.5)	57.9 (11.1)	*p* < 0.001
	18–64 years	440 (81.2%)	208 (88.1%)	147 (77.8%)	85 (72.6%)	
	≥65 years	102 (18.8%)	28 (11.9%)	42 (22.2%)	32 (27.4%)	
Country	France	124 (22.9%)	54 (22.9%)	50 (26.5%)	20 (17.1%)	
	Germany	120 (22.1%)	60 (25.4%)	36 (19.0%)	24 (20.5%)	
	Italy	124 (22.9%)	59 (25.0%)	35 (18.5%)	30 (25.6%)	
	Spain	120 (22.1%)	55 (23.3%)	40 (21.2%)	25 (21.4%)	
	UK	54 (10.0%)	8 (3.4%)	28 (14.8%)	18 (15.4%)	
Time since diagnosis	Median (IQR) months	33.9 (51.3)	27.5 (42.6)	39.8 (55.9)	46.5 (52.5)	*p* = 0.003
CIDP type	Typical CIDP CIDP variant	367 (67.7%) 175 (32.3%)	153 (64.8%) 83 (35.2%)	132 (69.8%) 57 (30.2%)	82 (70.1%) 35 (29.9%)	*p* = 0.452

CIDP: chronic inflammatory demyelinating polyradiculoneuropathy; *N*: sample size; SD: standard deviation; IQR: interquartile range.

### 3.2 Treatment and monitoring assessments

The distribution of maintenance and rescue/acute treatments is presented in Table 2. The proportion of patients prescribed treatment at the time of the survey increased with higher disability levels (mild: 78.3%; moderate: 89.9%; severe: 92.3%; *p* < 0.001). Intravenous immunoglobulin (mild: 40.5%, moderate: 51.8%, severe: 50.9%) and oral corticosteroids (mild: 47.0%, moderate: 35.9%, severe: 37.3%) were the most commonly prescribed treatment types across disability levels. The proportion of patients receiving both immunoglobulin and corticosteroids at the time of the survey was 9.7, 13.8, and 15.4% for mild, moderate and severe disability, respectively. A more detailed distribution of maintenance treatments is presented in Supplementary Table A2. Reasons for not receiving treatment, treatment history, and use and distribution of rescue/acute treatments are detailed in Supplementary Table A3. Among patients who were not receiving treatment at the time of the survey, the majority had previously been prescribed treatment for CIDP (mild: 52.9%, moderate: 78.9%, severe: 77.8%). Among patients not on treatment, the most commonly reported reasons for not receiving treatment were disease stability without treatment (mild: 82.4%, moderate: 73.7%, severe: 33.3%) and the patient not wanting medication (mild: 25.5%, moderate: 15.8%, severe: 55.6%). While most patients were receiving first-line treatment, the proportion of those on second-line or subsequent treatments rose with disability level (mild: 30.3%; moderate: 40.0%; severe: 42.6%; *p* = 0.008). Furthermore, the use of rescue treatment, both at the time of the survey and before, increased with increasing disability level (mild: 24.6%, moderate: 31.7%, severe: 43.6%, *p* = 0.002). Intravenous immunoglobulin and high dose steroids were the most administered kinds of rescue treatments for all disability levels.

**Table 2:** Maintenance treatment and rescue/acute treatments.

Treatment characteristic	Total (*N* = 542)	Mild disability (*N* = 236)	Moderate disability (*N* = 189)	Severe disability (*N* = 117)	*p*-value
Prescribed maintenance treatment at the time of the survey	463 (85.4%)	185 (78.3%)	170 (89.9%)	108 (92.3%)	*p* < 0.001
Distribution of treatment types*	(*n* = 463)	(*n* = 185)	(*n* = 170)	(*n* = 108)	
Intravenous Immunoglobulin (IVIg)	218 (47.1%)	75 (40.5%)	88 (51.8%)	55 (50.9%)	
Subcutaneous Immunoglobulin (SCIg)	37 (8%)	15 (8.1%)	11 (6.5%)	11 (10.2%)	
Corticosteroids (oral)	188 (40.6%)	87 (47.0%)	61 (35.9%)	40 (37.3%)	
Corticosteroids (IV)	30 (6.5%)	7 (3.8%)	14 (8.2%)	9 (8.3%)	
Immunosuppressants	14 (16.0%)	19 (10.3%)	36 (21.2%)	19 (17.6%)	
Biologics	52 (11.2%)	18 (9.7%)	21 (12.4%)	13 (12%)	
Other	48 (10.4%)	13 (7.0%)	17 (10.0%)	18 (16.7%)	
Line of treatment*	(*n* = 463)	(*n* = 185)	(*n* = 170)	(*n* = 108)	
1st line	293 (63.3%)	129 (69.7%)	102 (60.0%)	62 (57.4%)	
2nd line	140 (30.2%)	51 (27.6%)	58 (34.1%)	31 (28.7%)	
3rd line or higher	30 (6.5%)	5 (2.7%)	10 (5.9%)	15 (13.9%)	*p* = 0.008

* Among patients prescribed treatment at the time of the survey.

Line of treatment: sequence of therapies (first-line = initial, second-line = subsequent, etc.). *N* or *n*: sample size; IV: intravenous administration.

Electromyograms and nerve conduction tests were the most frequently used examinations for monitoring patients’ health [54.4% of patients; mean (SD) = 0.74 (0.86) tests/year], followed by complete blood count [39.7%; 1.13 (1.74)], renal function testing [25.2%; 0.65 (1.41)], and liver function testing [23.0%; 0.60 (1.41)]. The 10-Metre Walk Test was the most applied functional scale [20.9%; 0.43 (1.05)]. Test use was similar across disability levels (Supplementary Table S1).

### 3.3 Informal caregiver burden

**Figure 1:**
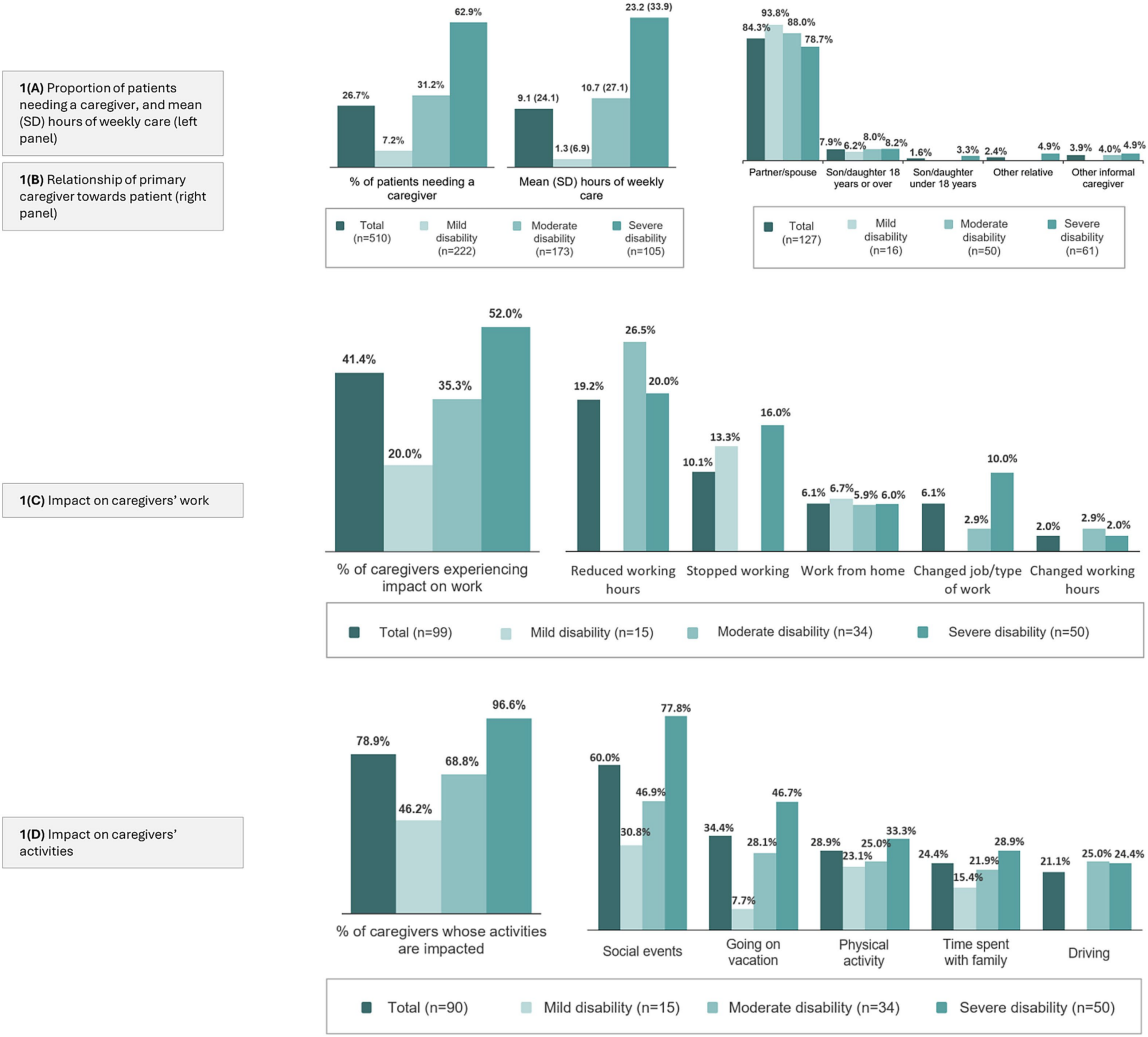
Informal caregiver burden. **(A)** Distribution of patients needing a caregiver and the hours of weekly care needed, **(B)** Relationship of primary caregiver towards patients, **(C)** Impact on the work of the primary caregiver and **(D)** Impact on activities of the primary caregiver. *n*: sample size; SD: standard deviation.

Informal caregiver burden is presented in Figures 1A–D; these data are also available in more detail in Supplementary Table S2. As shown in Figure 1A, the proportion of patients requiring informal (unpaid) care increased significantly with disability (mild: 7.2%; moderate: 31.2%; severe: 62.9%; *p* < 0.001). There were no statistically significant differences in hours of informal care per week between disability levels after controlling for age and time since diagnosis (mild: 1.3, moderate: 10.7, severe: 23.2, *p* = 0.075). In most cases, the primary caregiver was the patient’s partner or spouse (mild: 93.8%; moderate: 88.0%; severe: 78.7%; Figure 1B). In the overall sample, 26.7% of patients required a caregiver, with a mean of 9.1 h of care per week (SD: 24.1). The partner or spouse was the primary caregiver in 84.3% of cases, and caregiving affected the caregiver’s work and daily activities in 41.4 and 78.9% of cases, respectively.

Figure 1C shows that the impact of CIDP on the work of the primary caregiver increased with disability severity (*p* = 0.023). A work-related impact was reported in 20.0, 35.3, and 52.0% of caregivers for patients with mild, moderate, and severe disability, respectively. The most reported work-related impacts were reduced working hours (mild: 0.0%; moderate: 26.5%; severe: 20.0%) and cessation of work (mild: 13.3%; moderate: 0.0%; severe: 16%). Similarly, the impact of CIDP on caregivers’ daily activities increased with disability severity (*p* = 0.015; Figure 1D). An impact was reported in 46.2, 68.8, and 96.6% of caregivers for mild, moderate, and severe disability, respectively. The most frequently affected activities were participation in social events (mild: 30.8%; moderate: 46.9%; severe: 77.8%) and the ability to go on vacation (mild: 7.7%; moderate: 28.1%; severe: 46.7%).

The proportion of patients requiring help from a professional caregiver was 3.7, 3.7, and 51.9% among patients with mild, moderate, and severe disability, respectively. Professional caregivers provided on average 23.4 (SD 26.7) hours of care per week.

### 3.4 Hospitalizations

**Figure 2:**
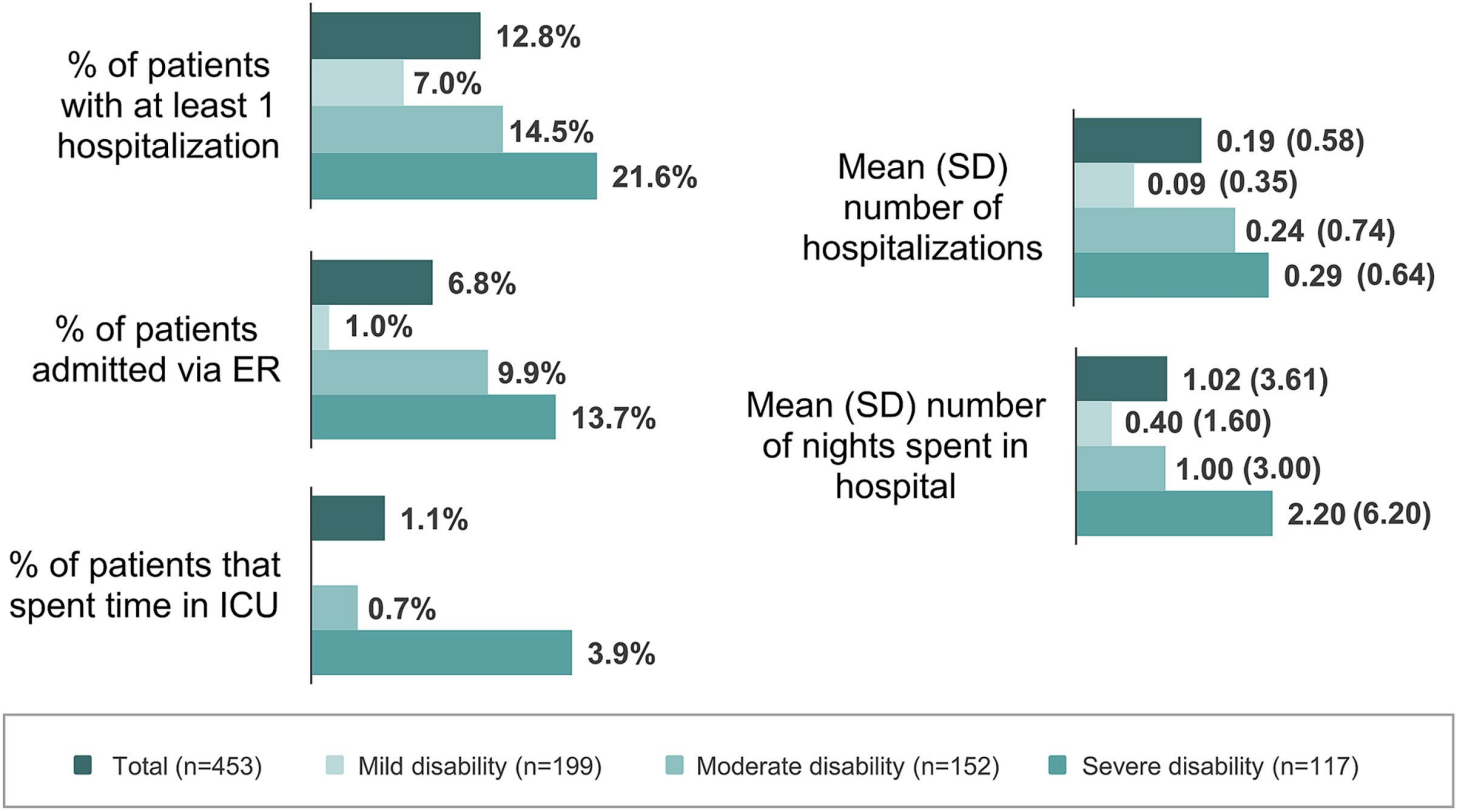
Distribution of hospitalizations, nights spent in hospital, ER admissions, and ICU stays. ER: emergency room; ICU: intensive care unit; *n*: sample size; SD: standard deviation.

Hospitalization results are presented in Figure 2; these data are also available in more detail in Supplementary Table S3. The proportion of patients with at least one hospitalization in the 12 months prior to the survey increased significantly with disease severity (mild: 7.0%; moderate: 14.5%; severe: 21.6%; *p* < 0.001). A similar trend was observed for hospitalizations through the emergency room (mild: 1.0%; moderate: 9.9%; severe: 13.7%; *p* < 0.001) and for those involving an ICU stay (mild: 0.0%; moderate: 0.7%; severe: 3.9%; *p* = 0.001). The average number of nights spent in the hospital also increased with disability severity (mild: 0.4 nights; moderate: 1.0; severe: 2.2; *p* < 0.001).

### 3.5 Use of mobility/supportive aids and home modifications

**Figure 3:**
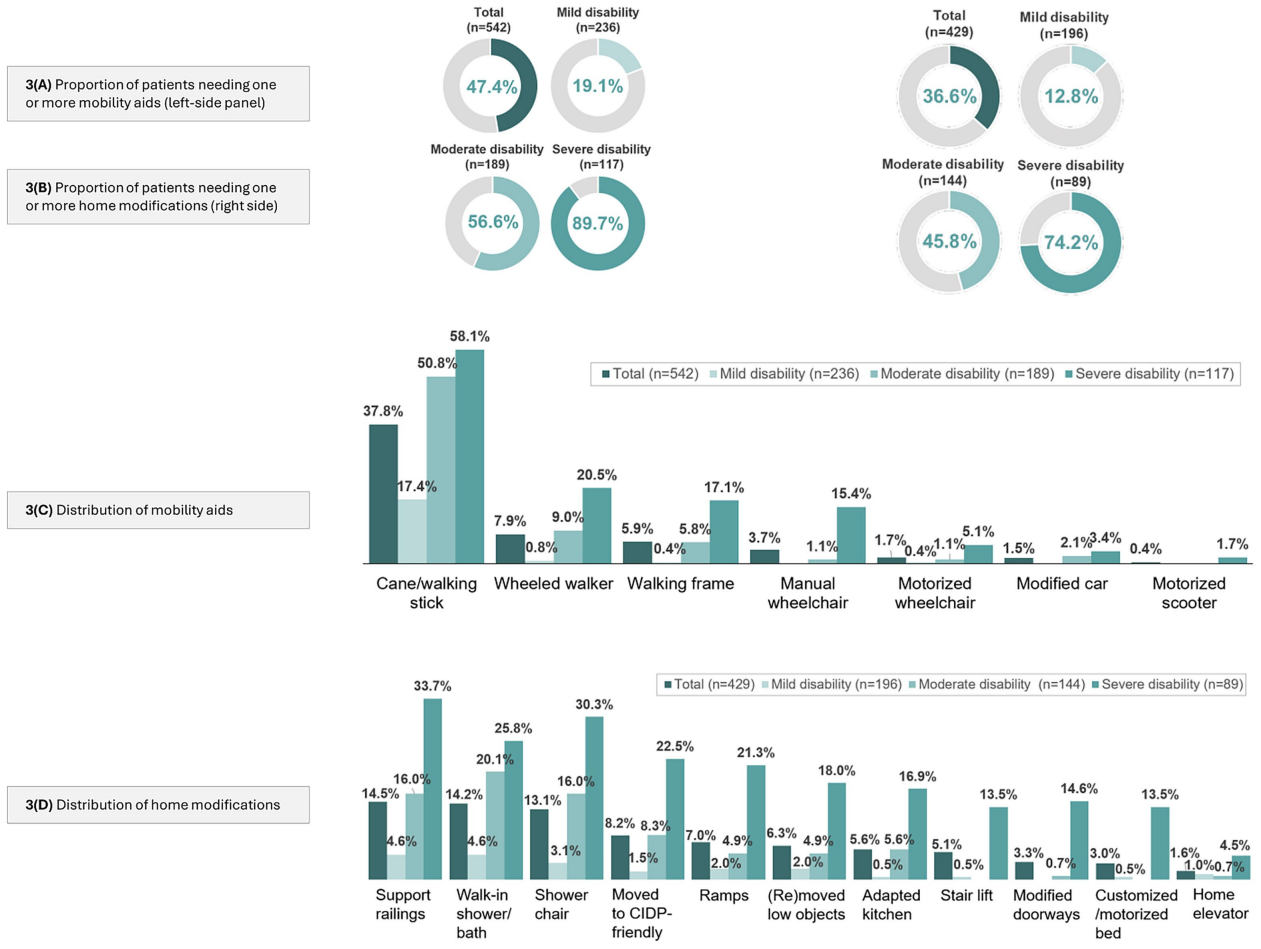
Mobility aid and home modification usage. **(A)** Proportion of patients using one or more mobility aids, **(B)** Proportion of patients using one or more home modifications, **(C)** Distribution of different mobility aids, **(D)** Distribution of different home modifications. *n*: sample size.

The use of mobility aids and the need for home modifications are presented in Figures 3A–D; these data are also available in more detail in Supplementary Table S4. The proportion of patients requiring one or more mobility aids increased with disability severity (mild: 19.1%; moderate: 56.6%; severe: 89.7%; *p* < 0.001; Figure 3A). Figure 3C shows that canes or walking sticks were the most used mobility aids across all groups (mild: 17.4%; moderate: 50.8%; severe: 58.1%). Among patients with severe disability, a substantial proportion used a wheeled walker (20.5%), walking frame (17.1%), or manual wheelchair (15.4%).

Similarly, the proportion of patients requiring one or more home modifications increased with disability severity (mild: 12.8%; moderate: 45.8%; severe: 74.2%; *p* < 0.001; Figure 3B). As shown in Figure 3D, the most implemented modifications were grab bars or supporting rails (mild: 4.6%, moderate: 16%, severe: 33.7%), shower chairs (mild: 3.1%, moderate: 16%, severe: 30.3%) and walk-in showers or baths (mild: 4.6%, moderate: 20.1%, severe: 25.8%).

The patient questionnaire also included items on the use of mobility or supportive aids and home modifications. To evaluate whether neurologists accurately assessed these aspects, we quantified the level of agreement between patient and physician responses in the subset of patients who completed the questionnaire. For items related to mobility or supportive aids, patients and neurologists provided concordant responses in 97.2% of cases. For items related to home modifications, concordance was observed in 94.7% of cases.

### 3.6 Employment rates and productivity losses

**Figure 4:**
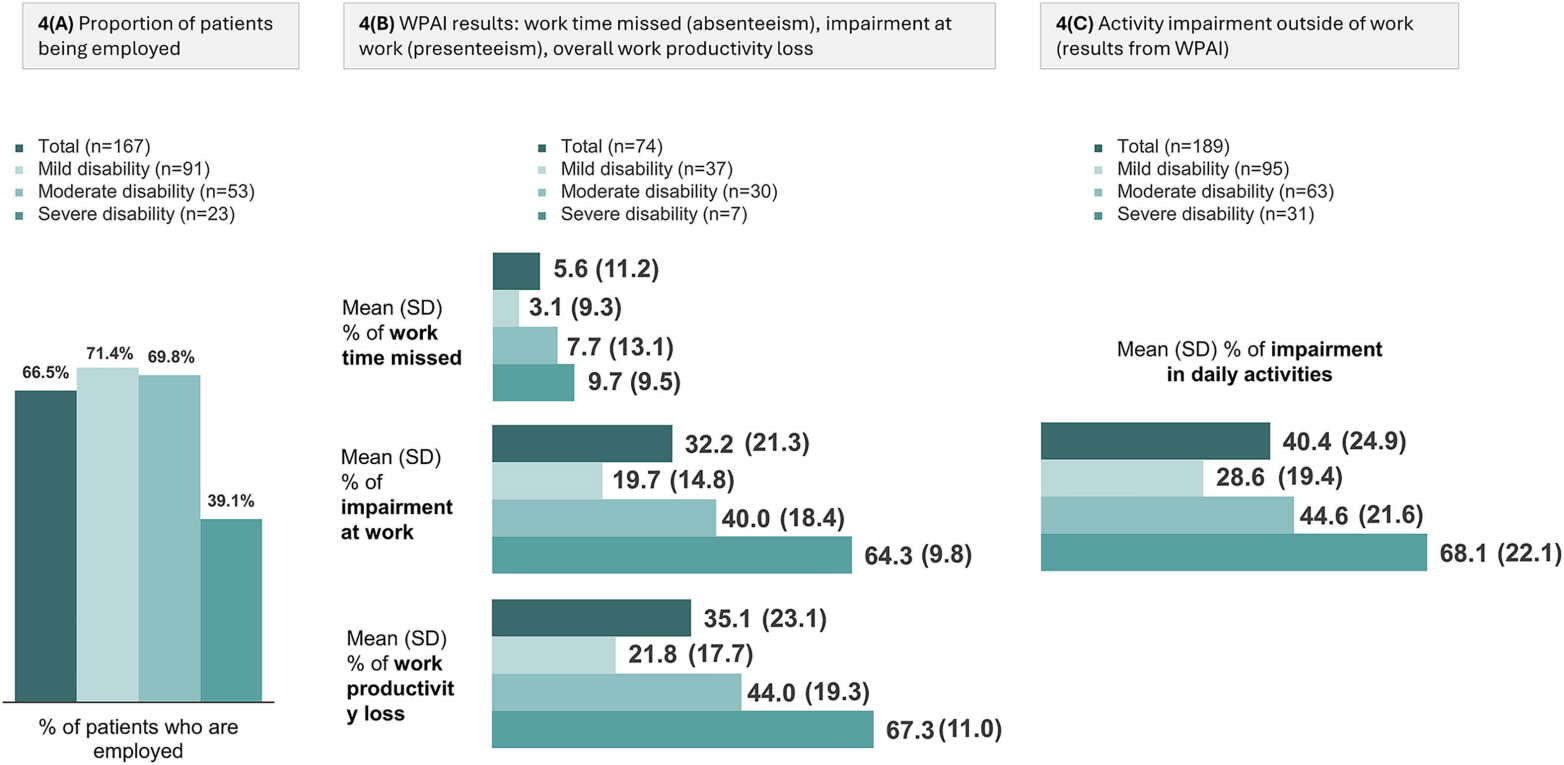
Employment rate and WPAI outcomes. **(A)** Percentage of patients who are employed, **(B)** Work productivity among patients who are employed, **(C)** Daily activity impairment. *n:* sample size; SD: standard deviation.

Patient-reported employment rates and work productivity outcomes among patients aged 18–64 years who completed the PSC are summarised in Figures 4A–C, with more detailed data provided in Supplementary Table S5. This subset comprised 93 (54.7%), 54 (31.8%), and 23 (13.5%) patients with mild, moderate, and severe disability, respectively.

As shown in Figure 4A, the proportion of patients employed at the time of the survey decreased with increasing disability severity (mild: 71.4%; moderate: 69.8%; severe: 39.1%; *p* = 0.049). Furthermore, employment rates were higher among men than among women for mild and moderate disease severity (mild: 86.3% versus 52.5%, moderate: 86.7% versus 47.8%, severe: 38.5% versus 40.0%). A more detailed breakdown of employment status for both patients aged 18–64 years and those aged ≥65 years is presented in Supplementary Table A4. Among patients aged ≥65 years, 89.3% were not working due to retirement. Among those aged 18–64 years, 52.7% were working full-time, 13.6% part-time, 13.0% were homemakers, 9.5% not working due to retirement, 6.5% unemployed, and 4.7% on long-term sick leave.

Results for the WPAI questions on absenteeism, presenteeism, and overall work productivity loss are presented for patients who were aged 18–64 years, employed and completed all relevant items (mild: 37 (49.3%); moderate: 30 (40%); severe: 7 (9.5%) patients; Figure 4B). Percentages of presenteeism and overall work productivity loss increased with disability severity (*p* < 0.001 for both). The mean percentage of work time missed was 3.1, 7.7, and 9.7% in the mild, moderate, and severe groups, respectively (*p* = 0.377). Work productivity was reduced by 19.7, 40.0, and 64.3%, respectively, with corresponding overall productivity losses of 21.8, 44.0, and 67.3%. Interpretation of results for patients with severe disability is limited by the smaller number of employed individuals in this subgroup.

Figure 4C shows that activity impairment outside of work also increased with disease severity (*p* < 0.001), with mean impairment scores of 28.6, 44.6, and 68.1% for patients with mild, moderate, and severe disability, respectively. Notably, all patients with severe disability reported some level of activity impairment.

### 3.7 Typical CIDP versus CIDP variants

The main outcomes for patients with typical CIDP and CIDP variants are presented in Supplementary Table S6. Patients with typical CIDP and CIDP variants had comparable levels of disability (mean INCAT score: 3.1 vs. 3.0). However, patients with typical CIDP were more frequently prescribed maintenance therapy (89.6% vs. 76.6%), more often required a caregiver (30.7% vs. 19.9%), and had lower employment rates (60.1% vs. 81.8%).

## 4 Discussion

This study offers a comprehensive assessment of HCRU, informal caregiver burden, and work productivity losses among patients with CIDP, stratified by disability level, based on data from a large, international European survey.

The cohort was predominantly male and largely comprised patients with typical CIDP across all disability levels, with mild disability being the most common. Increasing disability severity was associated with older age and longer time since diagnosis, supporting the characterization of CIDP as a progressive disease. The proportion of patients prescribed treatment at the time of the survey increased with disability level, with immunoglobulins and corticosteroids being the most frequently prescribed therapies, consistent with the European Academy of Neurology/Peripheral Nerve Society guidelines (3). Immunoglobulins were most commonly prescribed among patients with moderate and severe disability, whereas corticosteroids were more frequently prescribed in patients with mild disability, potentially reflecting neurologists’ prescribing patterns. In addition, the use of rescue therapy, both at the time of the survey and before, increased with disability severity. Overall, approximately one-third of patients had received rescue treatment at some point, highlighting the relapsing nature of CIDP. Unexpectedly, the majority of patients underwent electromyography and nerve conduction studies within the 12 months preceding the survey for CIDP monitoring, despite current guidelines recommending these investigations primarily for diagnosis rather than follow-up.

The findings confirm that CIDP imposes a substantial burden, not only on patients, but also on informal caregivers and society more broadly. Among employed patients, work productivity losses increased with disability severity. Greater disability was also associated with a higher need for caregiver support, which in turn contributed to disruptions in caregivers’ professional and personal lives. Hospitalization rates rose with increasing disability, as did the use of mobility aids and home modifications, reflecting the progressive functional decline associated with more severe disease. Overall, more than one third of patients reported having made adaptations to their home, with prevalence ranging from 13% among patients with mild disability to 74% among those with severe disability. Home modifications can be costly, and the need for such adaptations may therefore impose a substantial burden on patients. Consequently, the reported percentages may underestimate the true need for home modifications, as some patients may be unable to afford them.

To our knowledge, this is the first study reporting the caregiver burden for patients with CIDP in Europe. The findings do reinforce, however, the substantial impact of CIDP on economic productivity and workforce participation, which is in line with previous studies (4–6, 8, 10). In an online survey of 595 individuals with self-reported CIDP (80% based in the USA), 44% reported discontinuing work due to their symptoms, and 24% reported having to relocate to a new home (6). A cost-of-illness study conducted in southeast England found that patients with CIDP missed an average of 9 workdays per year due to their condition, and 32% had retired early (8). Similarly, a study of 108 patients in Germany reported that 14% were prematurely retired, 2% were unemployed, and 5% had reduced their working hours. Moreover, 24% of patients required inpatient care within a 3-month period (10). Collectively, these findings underscore the importance of timely diagnosis and effective management strategies to minimize functional decline, alleviate caregiver strain, and support patients’ ability to remain active and independent.

This study has several limitations. First, the patient sample may not fully represent the broader CIDP population, as patients who consult health care providers more frequently are more likely to be included. Nonetheless, we believe the observed associations in this study remain valid and informative, as the direction and strength of the relationships are unlikely to be substantially influenced by this potential overrepresentation. Furthermore, the average INCAT score in our sample (mean = 2.8, SD = 1.9) was comparable to values reported in the literature (e.g., Mengel et al. (10): mean = 3.3 SD = 2.1; Wonink et al. (19): median = 3, IQR = 4). These similarities suggest that our study population is reasonably representative of the broader CIDP population with respect to disease severity. Second, the cross-sectional nature of the survey captures only a snapshot in time and cannot fully represent the burden of a chronic, fluctuating condition like CIDP. Third, excessive missing data for some components (e.g., healthcare visits) prevented meaningful analysis and full cost-of-illness estimation. Finally, of the 542 patients for whom a PRF was completed, only 199 completed the DSP, leading to a relatively small sample for analysis of work productivity. The characteristics of this subset were generally comparable to those of the overall patient population, with the exception that it did not include patients from the UK and contained a higher proportion of women. Nevertheless, this real-world evidence provides valuable insights into HCRU in CIDP, particularly given the limited availability of large-scale data in this area. Future research would benefit from longitudinal designs that include all relevant cost components to more comprehensively assess the economic burden of CIDP. In addition, it would be valuable to explore other key determinants of socioeconomic burden, including country and healthcare reimbursement systems, as well as patient- and disease-related factors such as age, sex, household income, and disease duration.

These findings provide real-world evidence on the burden of illness in CIDP, highlighting areas of unmet need and supporting decision-making regarding resource allocation and treatment strategies. They also serve as valuable input for economic models evaluating the cost-effectiveness of treatments for CIDP.

## 5 Conclusion

This study highlights the substantial and multifaceted burden of CIDP, which increases with disease severity, highlighting the progressive functional decline associated with more severe disease. Beyond direct healthcare resource utilization, CIDP imposes considerable caregiver burden and productivity losses, underscoring the need for comprehensive disease management strategies aimed at preserving function and reducing the overall impact on patients, caregivers, and society.

## Data Availability

The data analyzed in this study is subject to the following licenses/restrictions: Not publicly available, but upon reasonable request and with permission of Adelphi Real World, access to the dataset can be granted. Requests to access these datasets should be directed to yasmin.taylor@omc.com.
