# Supplementary material for: Healthcare utilization and productivity losses associated with CIDP: results from an international survey
Source: Front Neurol. 2026 Jul 1;17:1846192. doi: 10.3389/fneur.2026.1846192 (PMC13368673; doi:10.3389/fneur.2026.1846192)
Supplement: Supplementary file 1 [file Supplementary_file_1.docx]

**Supplementary Material**

Healthcare utilization and productivity losses associated with CIDP: results from an international survey

**Appendix**

**Table A1.** Patient and disease characteristics for the subset of patients for whom patient-reported data is available (N=199)

|  |  | **Total**  **(N=199)** | **Mild disability**  **(N=103)** | **Moderate disability**  **(N=64)** | **Severe disability**  **(N=32)** | **p-value** |
| --- | --- | --- | --- | --- | --- | --- |
| **Sex** | Female  Male | 86 (43.2%)  113 (56.8%) | 44 (42.7%)  59 (57.3%) | 26 (40.6%)  38 (59.4%) | 16 (50%)  16 (50%) | p=0.675 |
| **Age** | Mean (SD) years | 52.4 (12.0) | 50.7 (11.0) | 52.5 (13.3) | 57.3 (11.6) | p=0.01 |
|  | 18 – 64 years | 170 (85.4%) | 93 (90.3%) | 54 (84.4%) | 23 (71.9%) |  |
|  | ≥ 65 years | 29 (14.6%) | 10 (9.7%) | 10 (15.6%) | 9 (28.1%) |  |
| **Country** | France | 32 (16.1%) | 17 (16.5%) | 13 (20.3%) | 2 (6.2%) | p=0.502 |
|  | Germany | 93 (46.7%) | 47 (45.6%) | 32 (50%) | 14 (43.8%) |  |
|  | Italy | 34 (17.1%) | 18 (17.5%) | 9 (14.1%) | 7 (21.9%) |  |
|  | Spain | 40 (20.1%) | 21 (20.4%) | 10 (15.6%) | 9 (28.1%) |  |
|  | UK | 0 (0%) | 0 (0%) | 0 (0%) | 0 (0%) |  |
| **Time since diagnosis** | Median (IQR) months | 32.6 (45.9) | 27.5 (35.3) | 39.6 (48.9) | 46.4 (50.3) | p=0.01 |
| **CIDP type** | Typical CIDP  CIDP variant | 139 (69.8%)  60 (30.2%) | 71 (68.9%)  32 (31.1%) | 44 (68.8%)  20 (31.2%) | 24 (75.0%)  8 (25.0%) | p=0.786 |

*CIDP: Chronic Inflammatory Demyelinating Polyradiculoneuropathy; N: sample size; SD: standard deviation; IQR: Interquartile Range*

**Table A2.** Detailed distribution of maintenance treatments

|  | **Total**  **(N=542)** | **Mild disability**  **(N=236)** | **Moderate disability**  **(N=189)** | **Severe disability**  **(N=117)** | **p-value** |
| --- | --- | --- | --- | --- | --- |
| Prescribed maintenance treatment at the time of the survey | 463 (85.4%) | 185 (78.3%) | 170 (89.9%) | 108 (92.3%) | p<0.001 |
| **Distribution of treatments*** | **(n=463)** | **(n=185)** | **(n=170)** | **(n=108)** |  |
| **Immunoglobulin (Ig)** |  |  |  |  |  |
| Intravenous Immunoglobulin (IVIg) | 218 (47.1%) | 75 (40.5%) | 88 (51.8%) | 55 (50.9%) |  |
| Subcutaneous Immunoglobulin (SCIg) | 37 (8%) | 15 (8.1%) | 11 (6.5%) | 11 (10.2%) |  |
| **Corticosteroids (oral)** |  |  |  |  |  |
| Prednisone/Prednisolone | 141 (30.5%) | 69 (37.3%) | 49 (28.8%) | 23 (21.3%) |  |
| Methylprednisolone | 34 (7.3%) | 11 (5.9%) | 10 (5.9%) | 13 (12%) |  |
| Dexamethasone | 13 (2.8%) | 7 (3.8%) | 2 (1.2%) | 4 (3.7%) |  |
| **Corticosteroids (IV)** |  |  |  |  |  |
| Methylprednisolone | 30 (6.5%) | 7 (3.8%) | 14 (8.2%) | 9 (8.3%) |  |
| **Immunosuppressants** |  |  |  |  |  |
| Azathioprine | 48 (10.4%) | 13 (7%) | 25 (14.7%) | 10 (9.3%) |  |
| Methotrexate | 6 (1.3%) | 3 (1.6%) | 2 (1.2%) | 1 (0.9%) |  |
| Tacrolimus | 8 (1.7%) | 1 (0.5%) | 6 (3.5%) | 1 (0.9%) |  |
| Mycophenolate mofetil | 11 (2.4%) | 3 (1.6%) | 2 (1.2%) | 6 (5.6%) |  |
| Cyclophosphamide | 2 (0.4%) | 0 (0%) | 1 (0.6%) | 1 (0.9%) |  |
| **Biologics** |  |  |  |  |  |
| Rituximab | 52 (11.2%) | 18 (9.7%) | 21 (12.4%) | 13 (12%) |  |
| **Other** |  |  |  |  |  |
| Gabapentin | 17 (3.7%) | 4 (2.2%) | 7 (4.1%) | 6 (5.6%) |  |
| Pregabalin | 10 (2.2%) | 4 (2.2%) | 3 (1.8%) | 3 (2.8%) |  |
| Plasmapheresis | 8 (1.7%) | 2 (1.1%) | 2 (1.2%) | 4 (3.7%) |  |
| Amitriptyline | 5 (1.1%) | 1 (0.5%) | 0 (0%) | 4 (3.7%) |  |
| Carbamazepine | 3 (0.6%) | 1 (0.5%) | 2 (1.2%) | 0 (0%) |  |
| Duloxetine | 5 (1.1%) | 1 (0.5%) | 2 (1.2%) | 2 (1.9%) |  |
| Venlafaxine | 2 (0.4%) | 0 (0%) | 1 (0.6%) | 1 (0.9%) | - |

*N or n: sample size; IV: intravenous administration*

**Table A3.** Reasons for not receiving treatment, treatment history, use and distribution of rescue/acute treatment

|  | **Total (N=542)** | **Mild disability**  **(N=236)** | **Moderate disability**  **(N=189)** | **Severe disability**  **(N=117)** | **p-value** |
| --- | --- | --- | --- | --- | --- |
| **Reason for not receiving any treatment*** | **(n=79)** | **(n=51)** | **(n=19)** | **(n=9)** |  |
| Patient’s condition is stable without treatment | 59 (74.7%) | 42 (82.4%) | 14 (73.7%) | 3 (33.3%) |  |
| Patient doesn’t want medication | 21 (26.6%) | 13 (25.5%) | 3 (15.8%) | 5 (55.6%) |  |
| Patient newly diagnosed and will be given treatment at next consultation | 5 (6.3%) | 2 (3.9%) | 2 (10.5%) | 1 (11.1%) |  |
| Patient not eligible for treatment | 2 (2.5%) | 1 (2.0%) | 0 (0.0%) | 1 (11.1%) |  |
| **Treatment history*** | **(n=79)** | **(n=51)** | **(n=19)** | **(n=9)** |  |
| Patient has previously been prescribed treatment for their CIDP | 49 (62.0%) | 27 (52.9%) | 15 (78.9%) | 7 (77.8%) |  |
| Patient has never received treatment for their CIDP | 30 (38.0%) | 24 (47.1%) | 4 (21.1%) | 2 (22.2%) |  |
| **Use of rescue/acute treatments** | **(n=542)** | **(n=236)** | **(n=189)** | **(n=117)** |  |
| Patient has never received rescue/acute treatment | 373 (68.8%) | 178 (75.4%) | 129 (68.3%) | 66 (56.4%) |  |
| Patient is currently not receiving rescue/acute treatment but has in the past | 129 (23.8%) | 48 (20.3%) | 47 (24.9%) | 34 (29.1%) |  |
| Patient is receiving rescue/acute treatment | 40 (7.4%) | 10 (4.2%) | 13 (6.9%) | 17 (14.5%) | p=0.002 |
| **Distribution of rescue treatments**** | **(n=40)** | **(n=10)** | **(n=13)** | **(n=17)** |  |
| Intravenous immunoglobulin | 27 (67.5%) | 7 (70%) | 10 (76.9%) | 10 (58.8%) |  |
| High dose steroids | 11 (27.5%) | 3 (30%) | 3 (23.1%) | 5 (29.4%) |  |
| Plasmapheresis | 3 (7.5%) | 0 (0%) | 0 (0%) | 3 (17.6%) |  |
| Immunoadsorption | 0 (0%) | 0 (0%) | 0 (0%) | 0 (0%) | - |

** among patients not prescribed treatment at the time of the survey*

*** among patients receiving rescue/acute treatment at the time of the survey*

*N or n: sample size; CIDP: Chronic Inflammatory Demyelinating Polyradiculoneuropathy*

**Table A4.** Employment status, as reported by the patient, for patients aged 18-64 years and patients aged ≥65 years.

|  | **Total (N=542)** | **Mild disability**  **(N=236)** | **Moderate disability**  **(N=189)** | **Severe disability**  **(N=117)** |
| --- | --- | --- | --- | --- |
| **Patients aged 18-64 years** | **(n=169)** | **(n = 92)** | **(n=54)** | **(n=23)** |
| Working full time | 89 (52.7%) | 55 (59.8%) | 28 (51.9%) | 6 (26.1%) |
| Working part time | 23 (13.6%) | 9 (9.8%) | 10 (18.5%) | 4 (17.4%) |
| Homemaker | 22 (13.0%) | 13 (14.1%) | 7 (13.0%) | 2 (8.7%) |
| Not working due to retirement | 16 (9.5%) | 8 (8.7%) | 5 (9.3%) | 3 (13.0%) |
| Unemployed | 11 (6.5%) | 4 (4.3%) | 2 (3.7%) | 5 (21.7%) |
| On long term sick leave | 8 (4.7%) | 3 (3.3%) | 2 (3.7%) | 3 (13.0%) |
| **Patients aged** ≥**65 years** | **(n=28)** | **(n=9)** | **(n=10)** | **n=(9)** |
| Not working due to retirement | 25 (89.3%) | 9 (100.0%) | 10 (100.0%) | 6 (66.7%) |
| Homemaker | 2 (7.1%) | 0 (0.0%) | 0 (0.0%) | 2 (22.2%) |
| Working full time | 1 (3.6%) | 0 (0.0%) | 0 (0.0%) | 1 (11.1%) |
