# Supplementary material for: Healthcare utilization and productivity losses associated with CIDP: results from an international survey
Source: Front Neurol. 2026 Jul 1;17:1846192. doi: 10.3389/fneur.2026.1846192 (PMC13368673; doi:10.3389/fneur.2026.1846192)
Supplement: Supplementary file 2 [file Table_1.DOCX]

***Supplementary Tables***

**Table S1:** Distribution of patients for which the test/assessment has been conducted to monitor the patient's CIDP, and mean (SD) number of times the test/assessment was conducted in the 12 months prior to the survey

|  | **Total**  **(N=531)** | **Mild disability**  **(N=233)** | **Moderate disability**  **(N=184)** | **Severe disability**  **(N=114)** |
| --- | --- | --- | --- | --- |
| **Muscle and nerve tests** |  |  |  |  |
| Electromyogram (EMG) and nerve conduction study | 289 (54.4%) 0.74 (0.86) | 119 (51.1%) 0.65 (0.77) | 106 (57.6%) 0.86 (0.97) | 64 (56.1%) 0.74 (0.83) |
| Nerve ultrasound | 27 (5.1%) 0.06 (0.31) | 7 (3%) 0.03 (0.2) | 12 (6.5%) 0.09 (0.41) | 8 (7%) 0.08 (0.3) |
| Somatosensory evoked potentials | 46 (8.7%) 0.12 (0.45) | 8 (3.4%) 0.05 (0.29) | 26 (14.1%) 0.21 (0.58) | 12 (10.5%) 0.13 (0.43) |
| Root stimulation tests | 35 (6.6%) 0.08 (0.3) | 8 (3.4%) 0.03 (0.18) | 15 (8.2%) 0.1 (0.35) | 12 (10.5%) 0.12 (0.38) |
| Neuromuscular junction evaluation | 33 (6.2%) 0.1 (0.47) | 7 (3%) 0.04 (0.23) | 17 (9.2%) 0.18 (0.71) | 9 (7.9%) 0.09 (0.31) |
| **Blood tests** |  |  |  |  |
| Complete blood count | 211 (39.7%) 1.13 (1.74) | 89 (38.2%) 1 (1.55) | 78 (42.4%) 1.27 (1.86) | 44 (38.6%) 1.19 (1.91) |
| C-reactive protein | 121 (22.8%) 0.57 (1.39) | 55 (23.6%) 0.53 (1.17) | 43 (23.4%) 0.66 (1.58) | 23 (20.2%) 0.52 (1.49) |
| Creatine kinase | 90 (16.9%) 0.47 (1.34) | 43 (18.5%) 0.44 (1.14) | 28 (15.2%) 0.48 (1.44) | 19 (16.7%) 0.52 (1.54) |
| Erythrocyte sedimentation rate | 89 (16.8%) 0.41 (1.22) | 37 (15.9%) 0.36 (1.04) | 33 (17.9%) 0.47 (1.33) | 19 (16.7%) 0.42 (1.35) |
| Fasting blood glucose | 103 (19.4%) 0.53 (1.35) | 43 (18.5%) 0.46 (1.27) | 36 (19.6%) 0.57 (1.31) | 24 (21.1%) 0.62 (1.57) |
| Glycosylated haemoglobin (HbA1c) | 71 (13.4%) 0.31 (1.01) | 31 (13.3%) 0.27 (0.83) | 24 (13%) 0.31 (0.94) | 16 (14%) 0.39 (1.38) |
| Liver function | 122 (23%) 0.6 (1.41) | 46 (19.7%) 0.49 (1.24) | 45 (24.5%) 0.65 (1.48) | 31 (27.2%) 0.74 (1.6) |
| Monoclonal gammopathy | 41 (7.7%) 0.18 (0.86) | 14 (6%) 0.14 (0.7) | 13 (7.1%) 0.22 (1.11) | 14 (12.3%) 0.21 (0.66) |
| Renal function | 134 (25.2%) 0.65 (1.41) | 57 (24.5%) 0.64 (1.45) | 49 (26.6%) 0.72 (1.54) | 28 (24.6%) 0.54 (1.09) |
| Serum protein electrophoresis (SPEP) | 46 (8.7%) 0.15 (0.64) | 21 (9%) 0.15 (0.67) | 15 (8.2%) 0.16 (0.66) | 10 (8.8%) 0.14 (0.51) |
| Vitamin B6 | 40 (7.5%) 0.23 (0.89) | 17 (7.3%) 0.21 (0.87) | 14 (7.6%) 0.24 (0.94) | 9 (7.9%) 0.24 (0.89) |
| Vitamin B12 | 57 (10.7%) 0.26 (0.91) | 25 (10.7%) 0.25 (0.9) | 19 (10.3%) 0.27 (0.94) | 13 (11.4%) 0.27 (0.9) |
| **Other tests** |  |  |  |  |
| Urine testing | 47 (8.9%) 0.16 (0.58) | 17 (7.3%) 0.11 (0.45) | 18 (9.8%) 0.17 (0.59) | 47 (8.9%) 0.16 (0.58) |
| **Scans and imaging** |  |  |  |  |
| MRI scan | 32 (6%) 0.07 (0.31) | 15 (6.4%) 0.08 (0.33) | 11 (6%) 0.08 (0.32) | 32 (6%) 0.07 (0.31) |
| **Scales and screening** |  |  |  |  |
| INCAT disability scale | 101 (19%) 0.39 (1.05) | 43 (18.5%) 0.44 (1.28) | 38 (20.7%) 0.37 (0.87) | 101 (19%) 0.39 (1.05) |
| Modified INCAT Sensory Sum scale (mISS) | 52 (9.8%) 0.2 (0.83) | 18 (7.7%) 0.22 (1.03) | 23 (12.5%) 0.19 (0.65) | 52 (9.8%) 0.2 (0.83) |
| Neuropathy Impairment Score | 50 (9.4%) 0.17 (0.62) | 19 (8.2%) 0.19 (0.72) | 21 (11.4%) 0.16 (0.52) | 50 (9.4%) 0.17 (0.62) |
| Inflammatory Rasch-built over all disability score (I-RODS) | 47 (8.9%) 0.18 (0.78) | 23 (9.9%) 0.24 (1.01) | 15 (8.2%) 0.14 (0.53) | 47 (8.9%) 0.18 (0.78) |
| MRC sum score | 100 (18.8%) 0.42 (1.15) | 50 (21.5%) 0.52 (1.34) | 35 (19%) 0.41 (1.12) | 100 (18.8%) 0.42 (1.15) |
| Grip strength assessment (vigorometer/handheld dynamometer) | 50 (9.4%) 0.18 (0.65) | 18 (7.7%) 0.17 (0.67) | 22 (12%) 0.21 (0.66) | 50 (9.4%) 0.18 (0.65) |
| 10-Metre Walk Test (10MWT) | 111 (20.9%) 0.43 (1.05) | 40 (17.2%) 0.44 (1.22) | 44 (23.9%) 0.42 (0.9) | 111 (20.9%) 0.43 (1.05) |
| Timed Up and Go test (TUG) | 51 (9.6%) 0.17 (0.65) | 10 (4.3%) 0.11 (0.66) | 28 (15.2%) 0.23 (0.64) | 51 (9.6%) 0.17 (0.65) |

*N: sample size; INCAT: Inflammatory Neuropathy Cause and Treatment score.*

**Table S2:** Distribution of patients needing a caregiver, relationship of primary caregiver towards patients and the impact on the work and activities of the primary caregiver

|  | **Total**  **(N=542)** | **Mild disability**  **(N=236)** | **Moderate disability**  **(N=189)** | **Severe disability**  **(N=117)** | **p-value** |
| --- | --- | --- | --- | --- | --- |
| **Caregiver** | **(n=510)** | **(n=222)** | **(n=173)** | **(n=105)** |  |
| n (%) of patients who need a caregiver | 136 (26.7%) | 16 (7.2%) | 54 (31.2%) | 66 (62.9%) | p<0.001 |
| **Hours of care per week** | **(n=510)** | **(n=222)** | **(n=173)** | **(n=105)** |  |
| Mean (SD) hours of total care per week | 9.1 (24.1) | 1.3 (6.9) | 10.7 (27.1) | 23.2 (33.9) | p=0.075 |
| **Relationship of primary caregiver towards patient*** | **(n=127)** | **(n=16)** | **(n=50)** | **(n=61)** |  |
| Partner/spouse | 107 (84.3%) | 15 (93.8%) | 44 (88.0%) | 48 (78.7%) |  |
| Son/daughter 18 years or over | 10 (7.9%) | 1 (6.2%) | 4 (8.0%) | 5 (8.2%) |  |
| Son/daughter under 18 years | 2 (1.6%) | 0 (0.0%) | 0 (0.0%) | 2 (3.3%) |  |
| Other relative | 3 (2.4%) | 0 (0.0%) | 0 (0.0%) | 3 (4.9%) |  |
| Other informal caregiver | 5 (3.9%) | 0 (0.0%) | 2 (4.0%) | 3 (4.9%) | - |
| **Impact on work of primary caregiver *** | **(n=99)** | **(n=15)** | **(n=34)** | **(n=50)** |  |
| **There is an impact on work** | **41 (41.4%)** | **3 (20%)** | **12 (35.3%)** | **26 (52%)** | p=0.023 |
| Caregiver stopped working | 10 (10.1%) | 2 (13.3%) | 0 (0%) | 8 (16%) |  |
| Caregiver reduced working hours | 19 (19.2%) | 0 (0%) | 9 (26.5%) | 10 (20%) |  |
| Caregiver changed job/ type of work | 6 (6.1%) | 0 (0%) | 1 (2.9%) | 5 (10%) |  |
| Caregiver works from home | 6 (6.1%) | 1 (6.7%) | 2 (5.9%) | 3 (6%) |  |
| Caregiver changed working hours | 2 (2%) | 0 (0%) | 1 (2.9%) | 1 (2%) |  |
| **Impact on activities of primary caregiver *** | **(n=90)** | **(n=13)** | **(n=32)** | **(n=45)** |  |
| **There is an impact on activities** | **71 (78.9%)** | **6 (46.2%)** | **22 (68.8%)** | **43 (96.6%)** | p=0.015 |
| Physical activity / exercise | 26 (28.9%) | 3 (23.1%) | 8 (25%) | 15 (33.3%) |  |
| Social events | 54 (60%) | 4 (30.8%) | 15 (46.9%) | 35 (77.8%) |  |
| Driving | 19 (21.1%) | 0 (0%) | 8 (25%) | 11 (24.4%) |  |
| Going on vacations / holidays | 31 (34.4%) | 1 (7.7%) | 9 (28.1%) | 21 (46.7%) |  |
| Time spent with family members | 22 (24.4%) | 2 (15.4%) | 7 (21.9%) | 13 (28.9%) |  |

* among caregivers

*N or n: sample size; SD: standard deviation*

**Table S3:** Distribution of hospitalizations, nights spent in hospital, ER admissions, and ICU stays

| **In the 12 months prior to the survey** | **Total**  **(N=542)** | **Mild disability**  **(N=236)** | **Moderate disability**  **(N=189)** | **Severe disability**  **(N=117)** | **p-value** |
| --- | --- | --- | --- | --- | --- |
| **Hospitalizations** | **(n=453)** | **(n=199)** | **(n=152)** | **(n=102)** |  |
| N (%) of patients having at least one hospitalization | 58 (12.8%) | 14 (7.0%) | 22 (14.5%) | 22 (21.6%) | p<0.001 |
| Mean (SD) number of hospitalizations | 0.19 (0.58) | 0.09 (0.35) | 0.24 (0.74) | 0.29 (0.64) | p<0.001 |
| Mean (SD) number of nights spent in the hospital | 1.02 (3.61) | 0.4 (1.6) | 1.0 (3.0) | 2.2 (6.2) | p<0.001 |
| **Hospitalizations through ER** | **(n=453)** | **(n=199)** | **(n=152)** | **(n=102)** |  |
| N (%) of patients having at least one hospitalization through ER | 31 (6.8%) | 2 (1.0%) | 15 (9.9%) | 14 (13.7%) | p<0.001 |
| **Hospitalizations including ICU stay** | **(n=453)** | **(n=199)** | **(n=152)** | **(n=102)** |  |
| N (%) of patients having at least one hospitalization including ICU stay | 5 (1.1%) | 0 (0.0%) | 1 (0.7%) | 4 (3.9%) | p=0.001 |

*N or n: sample size; SD: standard deviation; ER: emergency room; ICU: intensive care unit*

**Table S4:** Use of mobility aids and home modifications

|  | **Total**  **(N=542)** | **Mild disability**  **(N=236)** | **Moderate disability**  **(N=189)** | **Severe disability**  **(N=117)** | **p-value** |
| --- | --- | --- | --- | --- | --- |
| **Mobility aids** | **(n=542)** | **(n=236)** | **(n=189)** | **(n=117)** |  |
| **One or more mobility aids** | **257 (47.4%)** | **45 (19.1%)** | **107 (56.6%)** | **105 (89.7%)** | p<0.001 |
| Cane/walking stick | 205 (37.8%) | 41 (17.4%) | 96 (50.8%) | 68 (58.1%) |  |
| Wheeled walker | 43 (7.9%) | 2 (0.8%) | 17 (9%) | 24 (20.5%) |  |
| Walking frame | 32 (5.9%) | 1 (0.4%) | 11 (5.8%) | 20 (17.1%) |  |
| Manual wheelchair | 20 (3.7%) | 0 (0%) | 2 (1.1%) | 18 (15.4%) |  |
| Motorized wheelchair | 9 (1.7%) | 1 (0.4%) | 2 (1.1%) | 6 (5.1%) |  |
| Motorized scooter | 2 (0.4%) | 0 (0%) | 0 (0%) | 2 (1.7%) |  |
| Modified car (e.g. wheelchair accessible vehicle) | 8 (1.5%) | 0 (0%) | 4 (2.1%) | 4 (3.4%) |  |
| **Home modifications** | **(n=429)** | **(n=196)** | **(n=144)** | **(n=89)** |  |
| **One or more home modifications** | **157 (36.6%)** | **25** (**12.8%**) | **66** (**45.8%**) | **66** (**74.2%**) | p<0.001 |
| Moved to a more CIDP-friendly house (e.g. bungalow, ground-floor apartment) | 35 (8.2%) | 3 (1.5%) | 12 (8.3%) | 20 (22.5%) |  |
| Installed grab bars or support railings | 62 (14.5%) | 9 (4.6%) | 23 (16%) | 30 (33.7%) |  |
| Installed stair lift | 22 (5.1%) | 1 (0.5%) | 6 (4.2%) | 15 (16.9%) |  |
| Installed home elevator | 7 (1.6%) | 2 (1%) | 1 (0.7%) | 4 (4.5%) |  |
| Installed ramps | 30 (7%) | 4 (2%) | 7 (4.9%) | 19 (21.3%) |  |
| Removal/movement of low objects | 27 (6.3%) | 4 (2%) | 7 (4.9%) | 16 (18%) |  |
| Installed hospital-style or customized/motorized bed | 13 (3%) | 1 (0.5%) | 0 (0%) | 12 (13.5%) |  |
| Installed walk-in shower/bath | 61 (14.2%) | 9 (4.6%) | 29 (20.1%) | 23 (25.8%) |  |
| Uses a shower chair | 56 (13.1%) | 6 (3.1%) | 23 (16%) | 27 (30.3%) |  |
| Adapted kitchen | 24 (5.6%) | 1 (0.5%) | 8 (5.6%) | 15 (16.9%) |  |
| Modified doorways (e.g. widened) | 14 (3.3%) | 0 (0%) | 1 (0.7%) | 13 (14.6%) |  |

*N or n: sample size; CIDP: Chronic inflammatory demyelinating polyradiculoneuropathy*

**Table S5.**:Employment rates and WPAI outcomes among patients aged 18-64 years

|  | **Total**  **(N=170)** | **Mild disability**  **(N=93)** | **Moderate disability**  **(N=54)** | **Severe disability**  **(N=23)** | **p-value** |
| --- | --- | --- | --- | --- | --- |
| **Employment** | **(n=167)** | **(n=91)** | **(n=53)** | **(n=23)** |  |
| N (%) of patients who are employed | 111 (66.5%) | 65 (71.4%) | 37 (69.8%) | 9 (39.1%) | p=0.049 |
| **Absenteeism* (% work time missed)** | **(n=74)** | **(n=37)** | **(n=30)** | **(n=7)** |  |
| Patients with impairment, n (%) | 25 (33.8%) | 7 (18.9%) | 14 (46.7%) | 4 (57.1%) |  |
| Mean (SD), overall | 5.6 (11.2) | 3.1 (9.3) | 7.7 (13.1) | 9.7 (9.5) | p=0.377 |
| Mean (SD), among impaired | 16.6 (13.9) | 16.5 (16.3) | 16.6 (15.1) | 16.9 (4.2) |  |
| **Presenteeism* (% impairment while working)** | **(n=74)** | **(n=37)** | **(n=30)** | **(n=7)** |  |
| Patients with impairment, n (%) | 66 (89.2%) | 30 (81.1%) | 29 (96.7%) | 7 (100%) |  |
| Mean (SD), overall | 32.2 (21.3) | 19.7 (14.8) | 40 (18.4) | 64.3 (9.8) | p<0.001 |
| Mean (SD), among impaired | 36.1 (19.1) | 24.3 (12.5) | 41.4 (17.1) | 64.3 (9.8) |  |
| **Overall work productivity loss*** | **(n=74)** | **(n=37)** | **(n=30)** | **(n=7)** |  |
| Patients with impairment, n (%) | 66 (89.2%) | 30 (81.1%) | 29 (96.7%) | 7 (100%) |  |
| Mean (SD), overall | 35.1 (23.1) | 21.8 (17.7) | 44 (19.3) | 67.3 (11) | p<0.001 |
| Mean (SD), among impaired | 39.4 (20.7) | 26.9 (15.7) | 45.5 (17.8) | 67.3 (11) |  |
| **Activity impairment (% impairment in daily activities)** | **(n=189)** | **(n=95)** | **(n=63)** | **(n=31)** |  |
| Patients with impairment, n (%) | 173 (91.5%) | 80 (84.2%) | 62 (98.4%) | 31 (100%) |  |
| Mean (SD), overall | 40.4 (24.9) | 28.6 (19.4) | 44.6 (21.6) | 68.1 (22.1) | p<0.001 |
| Mean (SD), among impaired | 44.2 (22.7) | 34 (16.2) | 45.3 (21) | 68.1 (22.1) |  |

* among patients who are employed

*N or n: sample size; SD: standard deviation*

**Table S6:** Overview of outcomes for patients with Typical CIDP and CIDP variants

|  | **Typical CIDP** | **CIDP variant** |
| --- | --- | --- |
| **Sex** | **(n=367)** | **(n=175)** |
| Female | 134 (36.5%) | 71 (40.6%) |
| Male | 233 (63.5%) | 104 (59.4%) |
| **Age** | **(n=367)** | **(n=175)** |
| Mean (SD) years | 53.1 (12.1) | 55.9 (12.8) |
| **INCAT score** | **(n=367)** | **(n=175)** |
| Mean (SD) | 3.1 (1.9) | 3.0 (1.9) |
| **Maintenance treatment** | **(n=367)** | **(n=175)** |
| Prescribed maintenance treatment at the time of the survey | 329 (89.6%) | 134 (76.6%) |
| **Caregiver burden** | **(n=339)** | **(n=161)** |
| n (%) of patients who need a caregiver | 104 (30.7%) | 32 (19.9%) |
| Mean (SD) hours of total care per week | 9.9 (24.9) | 7.4 (22.3) |
| **Hospitalizations** | **(n=147)** | **(n=306)** |
| Mean (SD) number of hospitalizations | 0.2 (0.5) | 0.2 (0.6) |
| Mean (SD) number of nights spent in the hospital | 0.9 (3.8) | 1.2 (3.2) |
| **Mobility aids** | **(n=367)** | **(n=175)** |
| n (%) of patients needing one or more mobility aids | 175 (47.7%) | 82 (46.9%) |
| **Home modifications** | **(n=299)** | **(n=130)** |
| n (%) of patients needing one or more home modifications | 111 (37.1%) | 46 (35.4%) |
| **Employment** | **(n=123)** | **(n=44)** |
| n (%) of patients aged 18-64 who are employed | 75 (60.1%) | 36 (81.8%) |
